# Targeted Liposomal Chemotherapies to Treat Triple-Negative Breast Cancer

**DOI:** 10.3390/cancers13153749

**Published:** 2021-07-26

**Authors:** Yingnan Si, Ya Zhang, Hanh Giai Ngo, Jia-Shiung Guan, Kai Chen, Qing Wang, Ajeet Pal Singh, Yuanxin Xu, Lufang Zhou, Eddy S. Yang, Xiaoguang (Margaret) Liu

**Affiliations:** 1 Department of Biomedical Engineering, University of Alabama at Birmingham, 1825 University Blvd, Birmingham, AL 35294, USA; yingnan@uab.edu (Y.S.); yazhang9@uab.edu (Y.Z.); hanh96@uab.edu (H.G.N.); kaisdzb@uab.edu (K.C.); skylarqw@uab.edu (Q.W.); ajeeetts@uab.edu (A.P.S.); lfzhou@uab.edu (L.Z.); 2 Department of Medicine, University of Alabama at Birmingham, 703 19th Street South, Birmingham, AL 35294, USA; guan0926@uab.edu (J.-S.G.); yuanxin8@uab.edu (Y.X.); 3 Department of Radiation Oncology, University of Alabama at Birmingham, 1808 7th Avenue South, Birmingham, AL 35294, USA; shyang@uabmc.edu

**Keywords:** combined chemotherapies, targeted liposomes, triple-negative breast cancer

## Abstract

**Simple Summary:**

Triple-negative breast cancers (TNBCs) are mainly treated with standard chemotherapies. Combined therapies have been demonstrated as a promising treatment strategy in clinics. The aim of this study was to develop a new formulation of combined chemotherapies facilitated with a targeted delivery vehicle. We found that the mertansine and gemcitabine with different anti-cancer mechanisms resulted in high cytotoxicity in TNBC cells. The in vivo evaluations using two TNBC xenograft models confirmed the anti-tumor efficacy, i.e., significantly reduced tumor growth rate. Furthermore, the antibody-tagged liposomes effectively delivered the therapeutic drugs to TNBC tumor, which could reduce the side effects. This study is highly translational and the targeted liposomal drug formulation can be further investigated in future clinical trials for TNBC treatment.

**Abstract:**

Triple-negative breast cancers (TNBCs) are highly aggressive and recurrent. Standard cytotoxic chemotherapies are currently the main treatment options, but their clinical efficacies are limited and patients usually suffer from severe side effects. The goal of this study was to develop and evaluate targeted liposomes-delivered combined chemotherapies to treat TNBCs. Specifically, the IC_50_ values of the microtubule polymerization inhibitor mertansine (DM1), mitotic spindle assembly defecting taxane (paclitaxel, PTX), DNA synthesis inhibitor gemcitabine (GC), and DNA damage inducer doxorubicin (AC) were tested in both TNBC MDA-MB-231 and MDA-MB-468 cells. Then we constructed the anti-epidermal growth factor receptor (EGFR) monoclonal antibody (mAb) tagged liposomes and confirmed its TNBC cell surface binding using flow cytometry, internalization with confocal laser scanning microscopy, and TNBC xenograft targeting in NSG female mice using In Vivo Imaging System. The safe dosage of anti-EGFR liposomal chemotherapies, i.e., <20% body weight change, was identified. Finally, the in vivo anti-tumor efficacy studies in TNBC cell line-derived xenograft and patient-derived xenograft models revealed that the targeted delivery of chemotherapies (mertansine and gemcitabine) can effectively inhibit tumor growth. This study demonstrated that the targeted liposomes enable the new formulations of combined therapies that improve anti-TNBC efficacy.

## 1. Introduction

Triple-negative breast cancers (TNBCs) are the breast cancers that lack expression of estrogen receptor (ER), progesterone receptor (PR) and human epidermal growth factor receptor (HER)-2/neu. Currently chemotherapeutic agents are the most common clinical treatment strategies employed to suppress tumor growth, but TNBC patient responses differ from case to case. For instance, drug resistance due to drug efflux [1,2,3], apoptosis dysregulation [4,5], activation of survival, growth and invasion signaling pathways [6] or others [7,8] significantly limits their clinical efficacy and also leads to tumor recurrence and progression [9]. In addition, patients usually suffer from side effects, such as fatigue, emesis, hair loss, and anemia, due to a lack of an effective tumor targeting method.

The U.S. Food and Drug Administration (FDA) has approved liposomes as a drug delivery vehicle with guidance of “Chemistry, manufacturing, and controls; human pharmacokinetics and bioavailability; and labeling documentation” (FDA-2016-D-2817). The targeted liposomes have been developed and utilized to deliver drugs to the tumor or tumor microenvironment with minimal non-specific distribution in normal tissues or organs. Various tumor-targeting ligands, such as small molecules, oligonucleotides, peptides, monoclonal antibodies (mAbs) and antigen-binding fragments (Fabs), have been conjugated with liposomes. For example, the anti-epidermal growth factor receptor (EGFR) [10,11], HER2 [12] and vascular endothelial growth factor (VEGF) [13,14] antibodies or peptides have been linked to liposomal system to deliver doxorubicin or other medicines to breast cancers and other tumors. The fibronectin-mimetic peptide-PR_b [15], estrogen receptor-antagonist Tamoxifen [16] and peptide SP90 [17] have been used as linkers in liposomal drug formulation to treat breast cancers. Moreover, GAH mAb conjugated immunoliposomes have been fabricated to targeting deliver doxorubicin to treat human gastrointestinal cancers [18].

The EGFR, which stimulates the cancer proliferation via PI3K/RAS signaling, the repair of DNA damage and metastasis [19,20,21,22,23], is overexpressed in various tumors, e.g., TNBC (52–54%) [24,25], lung cancer (40%) [26,27], glioblastoma (50%), head and neck cancer (80–90%) [24,25,28], ovarian, cervical, bladder, gastric, endometrial and colorectal cancers [29]. EGFR is more predominant in TNBCs than other breast cancers [24,30], and usually correlates with tumor invasion and poor prognosis. From this perspective, anti-EGFR mAb was utilized in this study as a ligand to target TNBC. The targeted liposomal drug formulation is expected to prolong the circulation half-life and enhance the maximum tolerated dose.

Many innovative anti-cancer drugs failed in phase II clinical trials [31] although the pre-clinical results are promising. This could be attributed to the limitation of preclinical animal models such as lacking heterogeneity and tumor microenvironment. This challenge can be partially solved by applying patient-derived xenograft (PDX) models in the in vivo evaluation of the anti-tumor efficacy of new medicines. PDX models have been established by transplanting the cancerous cells or tissues from primary patient tumors and served as a good preclinical platform to predict the possible patient responses to new cancer medicine. This study used an EGFR-overexpressed PDX line to mimics the heterogeneity and tumor microenvironment of TNBC.

The objective of this study was to develop and validate a new liposomal drug formulation to treat TNBCs. The in vitro and in vivo evaluations of the EGFR mAb-Lipo-drugs (i.e., GC/DM1) showed high TNBC targeting and anti-tumor efficacy in cell lines and PDX xenograft mouse models. This study reported a new strategy to treat TNBCs, which has a great potential to be translated to clincial application in future.

## 2. Materials and Methods

### 2.1. Ethics Statement

All the animal studies performed in this research were conducted according to the Institutional Animal Care and Use Committee (IACUC) Protocol IACUC-21949 that was approved by the Institutional Biosafety Committee of University of Alabama at Birmingham. Female mice were used in this study because most breast cancers occur in women. To ensure similar health conditions and environmental factors, all mice were maintained in the same light, temperature and humidity-controlled space within the animal facility building.

### 2.2. Cell Lines and Media

The normal breast epithelium 184B5 cells (ATCC, Manssas, VA, USA, CRL-8799) and human TNBC cell lines MDA-MB-231 (ATCC, CRM-HTB-26), MDA-MB-468 (ATCC, Manassas, VA, USA, HTB-132) and MDA-MB-231-FLuc (GenTarget, San Diego, CA, USA, AC059-Puro) were maintained in DMEM/F12 medium supplemented with 4 g/L of glucose, 4 mM of L-glutamine, and 10% fetal bovine serum. The mouse TNBC 4T1 cells (ATCC, CRL-2539) were maintained in RPMI medium supplemented with 4 g/L of glucose, 4 mM of L-glutamine, and 10% fetal bovine serum. The seed cells were maintained in T-flasks at 37 °C and 5% CO_2_ in a humidified incubator (Caron, Marietta, OH, USA). All the media and biological reagents were purchased from Fisher Scientific (Waltham, MA, USA), unless otherwise specified.

### 2.3. TNBC Patient Tissue Microarray

Triple-negative breast cancer and normal tissue arrays were purchased from US Biomax (Derwood, MD, USA), which include 126 cases of breast carcinoma with ER^−^, PR^−^ and HER2^−^, and four cases of normal tissues. The primary rabbit anti-EGFR antibody and horseradish peroxidase (HRP)-conjugated secondary anti-rabbit antibody (Abcam, Cambridge, UK) were used to detect the EGFR expression. DAB substrate was utilized to visualize the positive staining. The images of TMA slide scanning were captured by Lionheart FX Automated Microscope (BioTek, Winooski, VT, USA). Gen5 software was used for the post-imaging processing.

### 2.4. Synthesis of Antibody-Liposome-GC/DM1

The 10 µmol of DOPC and 3.33 µmol of cholesterol (molar ratio = 3:1) were mixed in a round-bottom flask which contains 10 mL of chloroform. Then 0.8 µmol of DSPE-PEG-NHS linker, 0.67 µmol of DSPE-mPEG, 5 mg of GC, and 1 mg of DM1 were added into the mixture. To form a thin lipid film, the chloroform was evaporated by a rotary evaporator at 50 °C and 60 rpm for 1 hr. The gaseous chloroform residue was removed by vacuum pump. Then, the lipid was hydrated into 10 mL of phosphate buffered saline (PBS), followed by horizontal shaking at 37 °C and 120 rpm for 1 hr. The hydrated liposomes were sonicated in an ultrasonic bath (Fisher Scientific, Pittsburgh, PA, USA) for 15 min to reduce particle size. Then 6.67 nmol (1 mg) of anti-EGFR mAb was mixed with liposomes, followed by 2-h incubation at 40 °C. The packed drugs were titrated using HPLC (Shimadzu, Columbia, MD, USA) equipped with Shimadzu Nexcol C18 column (5 µm, 50 × 3.0 mm) with elution buffer of 55:45 methanol and water with flow rate of 0.65 mL/min at 25 °C.

### 2.5. Nanoparticle Tracking Analysis

The size distribution analysis and quantification of liposomes were conducted using NanoSight 300 (Malvern Panalytical Ltd., Malvern, UK) as previously reported [32,33]. Briefly, the synthesized liposomes were diluted with factors of 1:100 and 1:1,000 using PBS. Image capture was processed five times on each sample, 60 s per capture, with parameters of operation temperature 25 °C, pump perfusion rate 50, and detection threshold 9.

### 2.6. Transmission Electron Microscopy (TEM)

The liposomal size and shape were characterized by transmission electron microscopy following the previous approach [32]. Briefly, K100X Glow Discharge was utilized to discharge the formvar/carbon coated grids. The 10 µL of liposomes sample was mixed with 10 µL of water for dilution. Then, 10 µL of the mixture was dropped onto the grid and waited for one minute. To negatively stain the liposomes, 1% uranyl acetate was added to the grid. Images were captured on a Tecnai T12 transmission electron microscope (FEI, Hillsboro, OR, USA) equipped with an AMT CCD camera.

### 2.7. In Vitro Drug Cytotoxicity Study

The MDA-MB-231 and MDA-MB-468 cells were seeded into 96-well plates with viable cell density of 1 × 10^5^ cells/mL and viability of >95%. At 4 h post-seeding, the solution of free drugs (mertansine, gemcitabine, paclitaxel, or doxorubicin) was added to cells to reach a series of final concentration of 200, 100, 50, 20, 10, 5, 2, 1, 0 nM (DM1, GC, PTX, DM1 + GC, PTX + AC) and 0–1.5 µM (AC). At 5 days post-treatment, the viable cells were measured by Luminescent Cell Viability Assay (Promega, Madison, WI, USA). The relative viability was calculated as (viable cells in treatment group/control group) × 100%.

### 2.8. Western Blotting

The whole cell proteins were extracted using M-PER™ Mammalian Protein Extraction Reagent (Fisher, Waltham, MA, USA). The SDS-PAGE was performed by loading 20 µg of protein to 1.0-mm NuPAGE™ 4 to 12% Bis-Tris gels, then transferred to PVDF membrane using PowerEase^TM^ Touch Power Supply (Fisher). The blotted membrane was detected using primary mouse anti-human antibody, horseradish peroxidase (HRP)-conjugated secondary anti-mouse antibody (Abcam, Cambridge, UK), and Luminata Forte Western HRP substrate (Millipore, Boston, MA, USA). The SDS-PAGE gel and blotted membrane were imaged with MyECL imager (Thermo Scientific, Waltham, MA, USA) and quantified with ImageJ software (National Institutes of Health, Bethesda, MD, USA). Original western blots data is shown in File S1.

### 2.9. Live-Cell Confocal Microscopy

To label liposomes with fluorescent dye, 1,2-distearoyl-sn-glycero-3-phosphoethanolamine-N-(Cyanine 5) or PE-Cy5 was added into the lipid mixture during liposome fabrication. The MDA-MB-468 cells were seeded in a chambered glass coverslip (glass bottom) with a viable cell density of 1 × 10^5^ cells/mL. BacMam GFP transduction control reagent was added into culture medium with MOI of 50 to stain cytoplasm. NucBlue Live ReadyProbes reagent was added to stain nucleus following the operation manual. An amount of 2x10^9^ of anti-EGFR mAb-Lipo-Cy5 was added into the culture. After incubating overnight, the spent medium was replaced with fresh cell growth medium. Live-cell images were collected using Nikon A1R-HD25 confocal microscope with a high-speed resonance scanner (Nikon USA, Melville, NY, USA).

### 2.10. Flow Cytometry Analysis

The TNBC surface binding capability of EGFR mAb was evaluated in both human TNBC cell lines (MDA-MB-231 and MDA-MB-468), normal breast cell line (184B5) and mouse TNBC cell line (4T1) following our published methods [34,35,36]. Briefly, the anti-human EGFR mAb was labeled with Alexa Fluor™ 647 labelling kit (Life Technologies, part of Fisher, Carlsbad, CA, USA). Cells were trypsinized and collected from tissue culture flasks followed by centrifuging at 200× *g* for 5 min. Then, cells were resuspended into PBS plus 1% FBS with a density of 1 × 10^7^ cells/mL. To stain cells, 1 µg of AF647-EGFR mAb was mixed with 1 million of cells. The mixture was incubated at room temperature for 30 min. After three times of washing with PBS, the stained cells were analyzed by BD LSRII flow cytometer (BD Biosciences, San Jose, CA, USA).

### 2.11. Immunohistochemistry (IHC) Staining and Scoring

The paraffin-embedded slides were immersed in xylene twice for antigen retrieval. Then, the slides were placed in 3% H_2_O_2_ in PBS for 10 min at room temperature. A hydrophobic pen was used to surround the tissue with a hydrophobic barrier. The slides were blocked with 3% normal goat serum (same as secondary antibody host) in Triton X-100 in PBS for 1 h. After blocking, rabbit anti-human EGFR mAb (Abcam, Waltham, MA, USA) was added onto tissues with 1:100 dilution. After incubating overnight at 4 °C, slides were washed in PBS for 1 h. HRP-conjugated goat anti-rabbit IgG mAb was added onto tissues with 1:1000 dilution. After incubating at room temperature for 1 h, slides were washed with PBS for 3 times. DAB chromogen was dropped onto tissues for color development. Then, slides were counterstained with hematoxylin and dehydrated in absolute ethanol. Images were collected after drying out overnight.

As established in our previous study [34], ImageJ was used to score the IHC stained TNBC patient tissues and analyze the EGFR expression level. Briefly, the positive staining (red) and negative staining (blue) was quantified using RGB Measure function. The expression score of EGFR was calculated as (red_intensity_ − blue_intensity_)/blue_intensity_ × 100. The scoring criteria were defined as high expression with score of >10, medium expression with score of 7–10, and low expression with score of <7.

### 2.12. In Vivo Biodistribution and Ex Vivo Imaging

Five million of MDA-MB-231-FLuc cells were subcutaneously injected to the fat pad of each NSG (NOD.Cg-Prkdc^scid^ Il2rg^tm1Wjl^/SzJ) mouse. When the tumors reached > 100 mm^3^, mice were ready for biodistribution study. Then 15 µL of PE-Cy7 was added into the lipid mixture for fluorescent labeling during liposome fabrication. After 15-min bath sonication, 1 mg (6.67 nmol) of anti-EGFR mAb (for 3 mice) was mixed with the liposomes followed by 2-h incubation at 40 °C. The mAb-liposome-Cy7 was sterilized by 0.22 µm filters with PES membrane. Light green color was observed at the final step which indicates a successful labeling. About 200–250 µL of mAb-Lipo-Cy7 was intravenously (i.v.) injected into each mouse through the tail vein. Images were captured at 24 h post-injection by In Vivo Imaging System (Perkin Elmer, Waltham, MA) with the following parameters: excitation/emission = 710 nm/790 nm, exposure time = 5 s. Firefly luciferase substrate was i.p. injected into mice at 5 min pre-capture. After the live-animal imaging collection, mice were sacrificed. Tumor and main organs (brain, heart, lung, spleen and liver) were harvested for ex vivo imaging to further confirm the mAb specific targeting.

### 2.13. Cell Line-Derived Xenograft and In Vivo Treatment

Total of 1 × 10^7^ of MDA-MB-231-FLuc cells were subcutaneously injected to the right flank of each NSG female mouse. When tumors reached 50–100 mm^3^, mice were randomized into four groups: PBS, EGFR mAb-liposome (delivery vehicle), 12 mg/kg-BW of mAb-Lipo-DM1/GC, and 24 mg/kg of mAb-Lipo-DM1/GC (*n* = 5). Drugs/PBS/vehicle were i.v. administrated into tail vein on a Q4D × 4 schedule (4-day interval for 4 injections). The tumor size and mice body weight were measured every four days. Tumor volume was calculated as (width × width × length)/2 with dimension unit of millimeter. The mice were sacrificed when the tumor volume reached >1000 mm^3^ in control group.

### 2.14. Patient-Derived Xenograft (PDX) Model and In Vivo Treatment

The donor mice carrying TNBC PDX xenograft and host NSG mice were purchased from The Jackson Laboratory (Bar Harbor, ME, USA) and maintained at low passages of 2–4. When the tumor volume reached 2000–3000 mm^3^, mice were sacrificed to collect tumors. The harvested tumors were placed into a 100 × 15 mm petri dish, and minced into small fragments (<1 mm^3^) by a sterile blade. The minced tumor tissue was loaded into a 1-mL sterile syringe connected with a 16G needle (BD, Franklin Lakes, NJ, USA). About 40–50 µL of tumor tissue was subcutaneously injected into the right flank of each NSG mouse. After tumor was detected, mice were randomized into three groups. PBS, mAb-Lipo, 24 mg/kg mAb-Lipo-GC/DM1 were administrated into tail vein on a Q4D × 4 schedule (*n* = 4). Tumor volume was measured by a caliper once a week. Tumor volume was calculated as (width × width × length)/2 in millimeter with end point of volume >1000 mm^3^.

### 2.15. Statistical Analysis

All the quantitative data was shown as mean ± standard error of the mean (SEM). Two-tailed *t* test was utilized to test the statistical significance. One-way ANOVA and post hoc (Dunnett’s) analysis were conducted for data comparison. Statistical significance with *p* value of <0.005 was used for all tests.

## 3. Results

### 3.1. Construction and Characterization of Targeted Liposomes for Drugs Delivery

**Figure 1:**
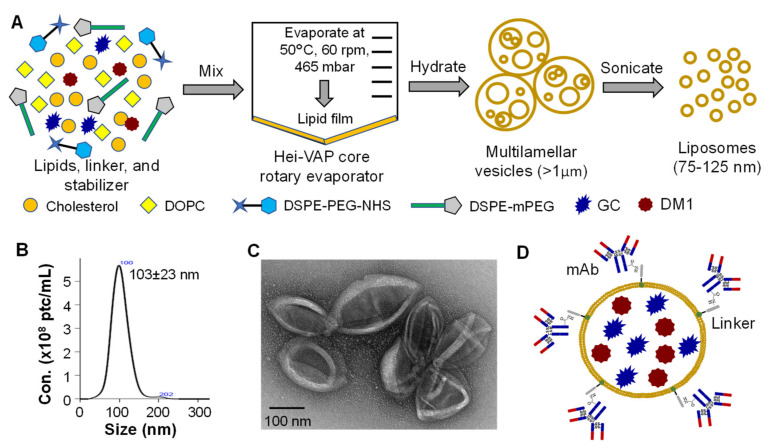
Liposome synthesis and characterization. (**A**) Liposome synthesis schematic diagram. The mixture of cholesterol, DOPC, DSPE-PEG-NHS and DSPE-mPEG was evaporated at 60 °C and 465 mbar for 2 h, followed by hydration to form multilamellar particles with diameter of >1 µm and sonication to generate homogeneous liposomes. (**B**) NanoSight showing homogenous distribution of liposomes with diameter of 103 ± 23 nm. (**C**) The transmission electron microscopes (TEM) image indicating the high purity and morphology of synthesized liposomes. (**D**) TNBC-targeting mAb was tagged to the surface of liposomes via the integrated DSPE-PEG-NHS linker and combined chemotherapies (i.e., GC and DM1) were packed into the mAb-Lipo.

Based on previously developed liposome synthesis procedure [37], we further optimized and constructed the targeted liposomal chemotherapies. In the optimal procedure, the DOPC and cholesterol were mixed together with DMPE-PEG-NHS that links the tumor-targeting antibody and liposomes, DSPE-mPEG that integrates into liposomes membrane to improve the circulation stability of liposomes [15,38,39], and the two chemotherapeutic drugs GC and DM1 (Figure 1A). This all-in-one construction process can increase the drug packaging rate to over 80% than that of 50% using incubation post liposomes synthesis. The synthesized Lipo-drugs were further purified using 300 kDa molecular weight cut-off (MWCO) PES concentrator to remove the unpacked free lipids, linker and drugs. In addition to loading therapies, the fluorescent dye, such as 18:0 Cyanine 7 (Cy7) which was used in this study, can also be encapsulated into liposomes to monitor the in vivo distribution of the delivered drugs facilitated with In Vivo Imaging System (IVIS).

The size distribution of synthesized Lipo-drugs was analyzed using NanoSight assay, demonstrating a homogenous distribution of the particles with an average diameter of 103 ± 23 nm (Figure 1B). The morphology was further characterized using transmission electron microscope (TEM) imaging, which showed that the Lipo-drugs had high purity with expected vehicle size (Figure 1C). In addition, we applied our developed surface tagging technology [32,33] to conjugate the anti-EGFR mAb to Lipo-drugs via DMPE-PEG-NHS linker to construct mAb-Lipo-drugs (Figure 1D), followed with purification and buffer exchange using 300 kDa MWCO PES concentrator. The constructed mAb-Lipo-drugs was used to deliver the combined therapies for TNBC treatment in this study.

### 3.2. In Vitro Anti-TNBC Cytotoxicity and Synergism Mechanisms

**Figure 2:**
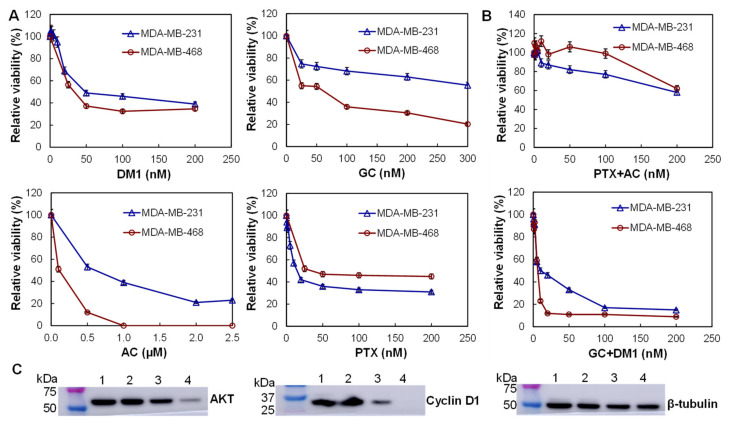
In vitro cytotoxicity and proliferation markers analysis. (**A**) The anti-TNBC cytotoxicity and dosage analysis of free drugs, i.e., mertansine (DM1), gemcitabine (GC), doxorubicin (AC), and paclitaxel (PTX), using human TNBC MDA-MB-231 cell line (**∆**) and MDA-MB-468 cell line (**○**) (data represent mean ± SEM, *n* = 3). (**B**) Cytotoxicity analysis of the combined chemotherapies, i.e., PTX + AC and GC + DM1, in TNBC MDA-MB-231 cell line (**∆**) and MDA-MB-468 cell line (**○**). (**C**) Western blotting analysis of proliferation and apoptosis biomarkers in MDA-MB-231 cells. 1: PBS control; 2: 5 nM GC and 20 nM DM1; 3: 20 nM GC and 50 nM DM1; 4: 200 nM GC and 200 nM DM1.

Four chemotherapies, including DM1 that inhibits the microtubule polymerization, GC that blocks DNA synthesis, AC that induces DNA damage, and PTX that defects mitotic spindle assembly, and their combinations were evaluated. The in vitro anti-cancer cytotoxicity of these drugs was tested. Multiple dosages of these drugs, including 0 (PBS), 0.5, 1, 2, 5, 10, 20, 50, 100, and 200 nM, were evaluated using both MDA-MB-231 and MDA-MB-468 cells. As presented in Figure 2A, DM1 and PTX showed high toxicity to both TNBC cell lines, and killed 60–70% cells at <50 nM. GC showed higher potency to MDA-MB-231 cells at dosages of 50–200 nM while AC was more toxic to MDA-MB-468 cells at dosages of 0.5–2.5 µM. The calculated IC_50_ values of DM1, GC, AC and PTX were 20, 12, 400, and 8 nM for MDA-MB-231 cells and 20, 100, 500, and 25 nM for MDA-MB-468 cells. Further evaluation showed that the combination of GC and DM1 (molar ratio of 1:1) killed 90% of MDA-MB-468 cells at dose of 20 nM and 85% of MDA-MB-231 cells at dose of 100 nM with IC_50_ of 5 nM for both cell lines (Figure 2B). The combination of PTX and GC killed 40% of TNBC cells at dose of 200 nM with IC_50_ of 50 nM for MDA-MB-231 and IC_50_ of 150 nM for MDA-MB-468 cells. These results indicated that the combination of DM1 and GC effectively reduced the TNBC cell growth in a dosage-dependent manner.

Western blotting analysis was performed to analyze the proliferation markers in the MDA-MB-231 cells treated with low (5 and 10 nM), medium (20 and 50 nM) and high (200 and 200 nM) doses of combined GC and DM1. It is found that the high and medium doses of GC/DM1 significantly reduced the relative expression of proliferation signaling proteins AKT to 0.86 and 0.26, respectively, from 0.96 (PBS, control), and the relative expression of cyclin D1 to 0.34 and 0.01, respectively, at 72 h post treatment (Figure 2C). The low dose had no obvious effect on the expression of these markers. These data indicated that the combination of GC and DM1 reduced proliferation of TNBC cells in a dose-dependent manner.

### 3.3. In Vitro Anti-TNBC Cytotoxicity and Synergism Mechanisms

**Figure 3:**
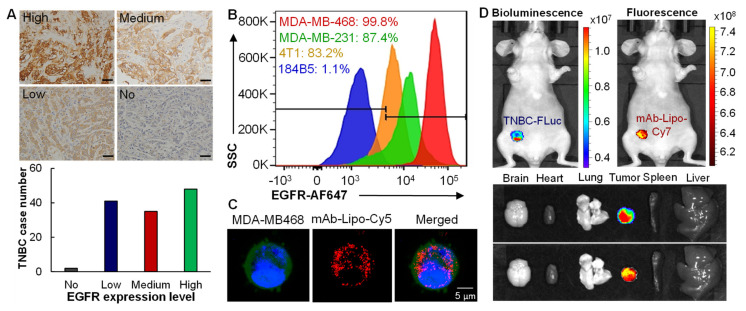
TNBC targeting by anti-EGFR mAb and mAb-Lipo. (**A**) The representative images of IHC staining to analyze the EGFR expression in TNBC patient tissue microarray. *n* = 126. Scale bar equals 50 µm. (**B**) Flow cytometry analysis showing that EGFR mAb has high surface binding rate to TNBC cells (MDA-MB-231 and MDA-MB-468) while low binding to normal breast cells (184B5). Cells were stained with 1 μg of mAb-AF647 per million cells at room temperature for 30 min. (**C**) Live-cell microscopy imaging of EGFR mAb-Lipo-Cy5. Whole cell labeled with GFP (displayed as green), nucleus labeled with NucBlue (blue), and mAb-Lipo labeled with PE-Cy5 (red). Scale bar equals 5 µm. (**D**) Live-animal IVIS imaging showing in vivo TNBC targeting by EGFR mAb-Lipo-Cy7 in MDA-MB-231-FLuc xenograft in NSG mice. Ex vivo images of tumor and important organs, including brain, heart, lung, spleen and liver, also confirmed tumor targeting of mAb. The mAb-Lipo-Cy7 was i.v. injected via tail vein (*n* = 3), and IVIS images were taken at 24 h post injection.

To assess the surface expression of EGFR receptor in TNBCs, the patient tissue microarray (TMA, *n* = 126) was stained using EGFR Ab. The representative IHC staining image and the relative expression were described and summarized in Figure 3A. The staining revealed that 38% (48/126) TNBC patients tissues had high expression with score of >10; 28% (35/126) tissues showed intermediate expression with score of 7–10; and 33% (41/126) tissues had low or minimal expression with score of <7 (Figure 3A). In addition, we found that the skin tissues had low EGFR expression but spleen tissues had high expression.

Flow cytometry analysis was performed in normal breast cells 184-B5 (negative control), human TNBC MDA-MB-231 and MDA-MB-468 cells, and mouse TNBC 4T1 cells to assess the in vitro TNBC-surface binding of EGFR mAb-AF647 at room temperature. We found that the surface binding rate was 87.4–99.8% in human TNBC cells, 83.2% in mouse TNBC cells, and 1.05% (minimal) in non-cancerous breast cells. The live-cell confocal microscopy imaging was performed to evaluate the capability of intracellular drug delivery. The results showed that mAb-Lipo-Cy5 (displayed as red) was internalized and located in cytoplasm (displayed as blue) after mixing with TNBC cells (Figure 3C). These data suggested that the EGFR mAb can direct the liposomes to target TNBC cells and deliver the fluorescent dye (Cy5).

The in vivo TNBC-targeting of Cy7-labeled mAb-Lipo was assessed in MDA-MB-231-FLuc xenograft mouse model. The live-animal IVIS imaging demonstrated that the Cy7 fluorescence overlapped with the FLuc bioluminescence at 24 h post i.v. injection of EGFR mAb-Lipo-Cy7, indicating that the mAb-Lipo preferentially bound to TNBC tumors (Figure 3D). Additonally, the ex vivo IVIS confirmed the TNBC xenograft-trageting by mAb-Lipo while no obvious binding to major organs such as brain, heart, lung, spleen and liver. Altogether, these data confirmed that the constructed mAb-Lipo can effectively target TNBC cells or tumor.

### 3.4. Tolerated Dosage in BALB/cJ

**Figure 4:**
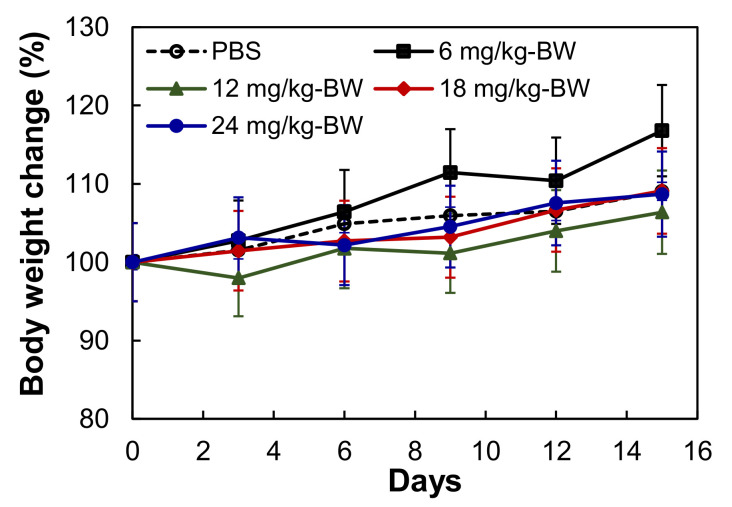
Dosage effect of mAb-Lipo-drugs on body weight of mice. PBS (**○**), 6 mg/kg (■), 12 mg/kg (▲), 18 mg/kg (♦), and 24 mg/kg (●). *n* = 3.

To investigate the tolerated dosage of mAb-Lipo-drugs, the non-tumor bearing BALB/cJ mice were treated with EGFR mAb-Lipo-GC/DM1 at dosages of 0, 6, 12, 18 and 24 mg/kg via i.v. injection. No obvious changes in water intake, breathing and locomotion, body weight, or overall survival were observed for two weeks after injection. The changes of body weight were in the range of 6.4–11.8% for all the groups (Figure 4). Two dosages, i.e., 12 and 24 mg/kg, were used in the in vivo anti-tumor efficacy study.

### 3.5. In Vivo Anti-tumor Efficacy in TNBC Cell Line Xenograft Model

**Figure 5:**
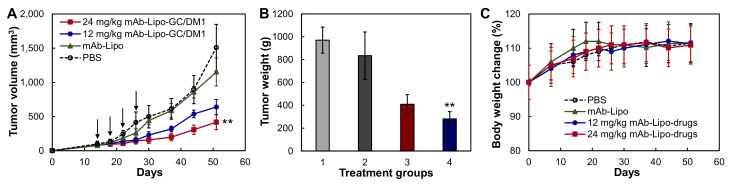
In vivo anti-TNBC efficacy of EGFR mAb-Lipo-drugs in MDA-MB-231-FLuc xenograft mouse models. (**A**) Tumor volume changes post treatment (data represent mean ± SEM, *n* = 5). PBS (**○**), drug delivering vehicle mAb-Lipo (▲), 12 mg/kg mAb-Lipo-GC/DM1 (●) and 24 mg/kg mAbs-Lipo-GC/DM1 (■). Tumor volume was measured with calipers, and calculated as ellipsoid. Black arrow indicating the i.v. injection of controls or therapies. (**B**) Wet weight of the tumors excised from euthanized mice. (**C**) The normalized body weight after treatment. ** *p* <0.005 vs. PBS using ANOVA followed by Dunnett’s *t*-test.

To evaluate the in vivo treatment efficacy of the targeted liposomal chemotherapies, we generated MDA-MB-231-FLuc xenograft models using 6-week female NSG mice. When tumor volume reached ~75 mm^3^, the xenografted mice were treated through i.v. administration of PBS (control), mAb-Lipo (delivery vehicle), 12 or 24 mg/kg-BW mAb-Lipo-GC/DM1 on a Q4D×4 schedule. As shown in Figure 5A, the TNBC tumor growth was significantly inhibited in the mAb-Lipo-GC/DM1 treatment groups as compared to PBS and mAb-Lipo control groups (*p* ≤ 0.005). The 12 mg/kg and 24 mg/kg of targeted liposomal GC/DM1 reduced tumor growth rate by 54% and 69%, respectively, as compared to PBS control group. It is clear that the targeted mAb-Lipo is an effective drug delivery vehicle for combined chemotherapies (i.e., conventional GC and highly potent DM1) for TNBC treatment. There was no obvious body weight difference among all the four groups (Figure 5B), indicating the overall therapeutic safety of the treatment.

### 3.6. In Vivo Anti-TNBC Efficacy in PDX Model

**Figure 6:**
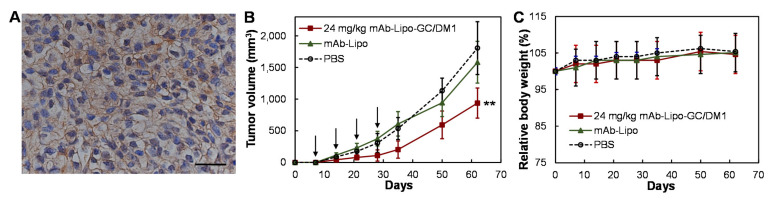
In vivo anti-TNBC efficacy of mAb-Lipo-drugs in PDX models. (**A**) Representative images of IHC staining of the PDX xenograft tissues showing strong EGFR surface expression in J000103634 line. Scale bar equals to 20 µm. (**B**) Tumor volume post treatment (*n* = 4). PBS (**○**), drug delivering vehicle mAb-Lipo (▲), and 24 mg/kg mAbs-Lipo-GC/DM1 (■). Arrow indicating the i.v. injection of control or therapies. (**C**) The normalized body weight after treatment. ** *p* <0.005 vs. PBS using ANOVA followed by Dunnett’s *t*-test.

Human TNBCs are heterogeneous and PDX models can recapitulate the heterogeneity and tumor microenvironment. We utilized TNBC PDX lines from Jackson Lab, confirmed the EGFR surface receptor expression in line of J000103634, and successfully passaged and propagated the PDX line in NSG mice. As shown in Figure 6A, the IHC staining of the PDX tissue section showed high surface expression of EGFR. Treated with PBS (negative control), mAb-Lipo (vehicle), 24 mg/kg-BW mAb-lipo-GC/DM1 on a Q4Dx4 schedule (*n* = 4), the tumor volume and body weight of PDX models were monitored once a week. It is clear that the group treated with targeted liposomal chemotherapies had 62–67% reduced PDX tumor volume than the control groups (Figure 6B). The body weight had no obvious change among different groups (Figure 6C).

## 4. Discussion

Chemotherapies are still the major strategy to treat TNBC in clinics. This research identified a new formulation of combined chemotherapies and also established a targeted delivery method for TNBC treatment, which could address the challenges of drug resistance or poor clinical efficacy as well as treatment related toxicities. In this study, we have evaluated a highly potent drug and several standard chemotherapies for cancer treatment, including DM1, GC, AC, and PTX [40], and two combinations of these drugs. Our results showed that combining standard GC and potent DM1 can kill over 90% TNBC cells with significantly reduced IC_50_ value and also effectively inhibit TNBC tumor growth in both cell line-derived xenograft models and patient-derived xenograft models. In addition to the improved cytotoxicity, GC and DM1 have different anti-cancer mechanisms so the combination could reduce the possibility of drug resistance development during long-term treatment compared to monotherapy. Therefore, the combination of GC and DM1 has great potential to treat TNBC.

We established and optimized the procedures of neutral liposomes synthesis to pack chemotherapies, surface tagging of TNBC-targeting antibody (mAb-Lipo), PEGylation, drugs packing, purification and characterization following the published guideline and protocols [41,42,43,44,45,46,47,48,49,50,51,52] with optimization. Non-targeting liposomes [53,54,55,56,57] have been used to deliver chemotherapies and other therapies, but the mAb-Lipo has multiple advantages, such as cancer-specific targeting, high packing capability with the developed all-in-one synthesis procedure, and high plasma stability and prolonged half-life with integrated PEG. Importantly, our surface tagging technology enables conjugating single or two (even multiple) antibodies to achieve dual-targeting to cover more patients with heterogeneous tumors. In addition to chemotherapies, the cationic liposomes encapsulated plasmid DNA (named as lipoplexes) have been evaluated in clinical trials for cystic fibrosis [58], non-small-cell lung cancer [59], metastatic melanoma [60,61], and epithelial ovarian, fallopian tube or primary peritoneal cancers [62] treatment.

Literature [25,63,64,65], clinical data [24,25,63] and our immunohistochemistry staining of patient tissue microarray show that EGFR is an excellent surface receptor in human [66,67,68] and mouse [69,70,71] TNBCs. For example, the anti-EGFR cetuximab and panitumumab are used in clinic to treat head and neck cancer [72,73,74] and colorectal cancer [75,76,77]. Moreover, the cetuximab mediates antibody-dependent cell cytotoxicity (ADCC) in the intratumoral space and primes adaptive and innate cellular immunity [78]. By tagging anti-EGFR mAb (cetuximab) to the surface of liposomes, we not only achieve TNBC tumor targeting but also could integrate the immunotherapy of the mAb. Of course, further investigation is needed to delineate the possible integrated anti-TNBC mechanisms of the tagged mAb and delivered GC and DM1 in future.

The TNBC xenograft models derived from various cell lines have been widely used in vivo to evaluate the tumor treatment efficacy. The PDX models are more advanced to evaluate new therapies as they have multiple advantages such as capturing TNBC heterogeneity and tumor microenvironment. For instance, PDX tumors can accurately recapitulate the phenocopy and mutation status of patient tumors, and resemble and maintain the biological behavior correlating with high metastasis, high heterogeneity and poor survival of TNBC patient tumors. Limited by the fresh patient tissues assessment and pathology analysis, many research labs have difficulty to establish in-house TNBC PDX models. We evaluated the Jackson lab commercial PDX lines and established a robust procedure to passage and maintain PDX lines in the research lab. The identified EGFR overexpressing PDX lines can be used as a good model to evaluate the therapeutic efficiency of newly developed therapies.

## 5. Conclusions

The combination of chemotherapies with different anti-cancer mechanisms (gemcitabine and mertansine in this study) has great potential to treat the highly aggressive TNBC. The technical challenges to apply combined chemotherapies, including circulation stability and side effects, can be overcome by the application of a targeted liposomal delivery vehicle. Importantly, different drug combinations can be easily adapted to this system for the treatment of recurrent cancer. Despite the promising results, the developed new formulation needs further evaluation in the future, such as pharmacokinetics, dosage optimization, metastatic tumor treatment and immune modulatory response.

## Data Availability

All data for this paper can be found in the text.

## Supplementary Materials

The following are available online at https://www.mdpi.com/article/10.3390/cancers13153749/s1, File S1: Original western blots data.
