# Supplementary material for: Targeted Liposomal Chemotherapies to Treat Triple-Negative Breast Cancer
Source: Cancers (Basel). 2021 Jul 26;13(15):3749. doi: 10.3390/cancers13153749 (PMC8345094; doi:10.3390/cancers13153749)
Supplement: Supplementary file 1 [file cancers-13-03749-s001.zip › cancers-1302059-SI.pdf]

cancers

MDPI

Supplementary Files

Targeted Liposomal Chemotherapies to Treat Triple-Negative Breast Cancer

Yingnan Si, Ya Zhang, Hanh Giai Ngo, Jia-Shiung Guan, Kai Chen, Qing Wang, Ajeet Pal Singh, Yuanxin Xu, Lufang Zhou, Eddy S. Yang and Xiaoguang "Margaret" Liu

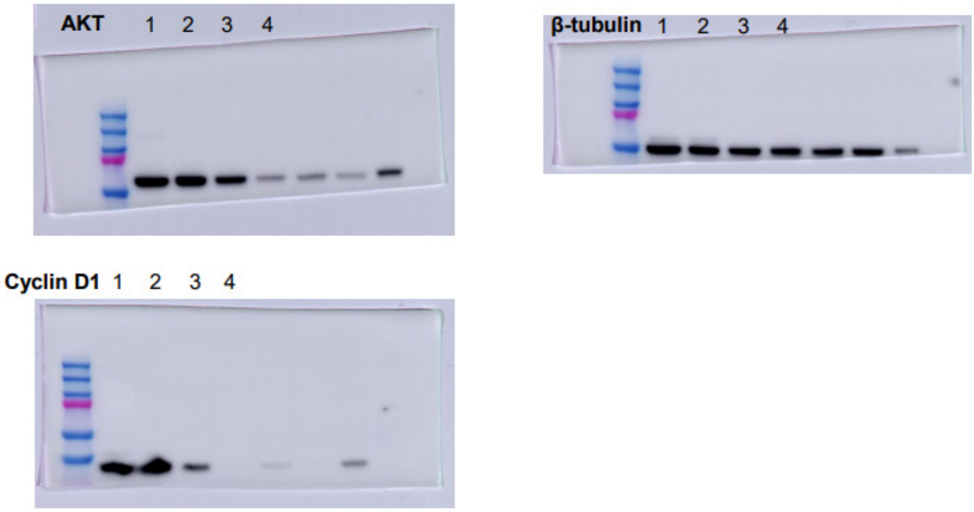

Figure S1. Original western blots data.

Cancers **2021**, *13*, 3749. https://doi.org/10.3390/cancers13153749 www.mdpi.com/journal/cancers
